# Effect of Increased Inoculum Size on Drug Susceptibility of *Mycobacterium tuberculosis*: Challenges in Reliable Drug Resistance Detection

**DOI:** 10.1002/mbo3.70345

**Published:** 2026-06-23

**Authors:** Kubra Yildirim, Cemilenur Atas, Ece Simsek, Mir Pooya Salehi Moharer, Meltem Uzun, Ahmet Yilmaz Coban

**Affiliations:** ^1^ Faculty of Health Sciences Research Laboratory, Tuberculosis Research Unit Akdeniz University Antalya Turkey; ^2^ Department of Nutrition and Dietetics, Faculty of Health Sciences Akdeniz University Antalya Turkey; ^3^ Department of Medical Biotechnology, Institute of Health Sciences Akdeniz University Antalya Turkey; ^4^ Private Akdeniz Sifa Hospital Antalya Turkey; ^5^ Department of Medical Microbiology, Istanbul Medical School Istanbul University Capa Istanbul Turkey

**Keywords:** drug susceptibility test, inoculum density, inoculum size, Mycobacterial inoculum, *Mycobacterium tuberculosis*, tuberculosis

## Abstract

Inoculum preparation of *Mycobacterium tuberculosis* isolates is a critical step for the reproducibility of drug susceptibility tests (DST) and the prevention of false results. In this study, we investigated the effect of increasing inoculum sizes of *M. tuberculosis* isolates on DST. For this purpose, primary drug susceptibilities of five ATCC strains and 24 *M. tuberculosis* isolates were determined by the proportion method on 7H10 agar using six different inoculum sizes (10^−2^ dilution of McFarland no 1 as reference inoculum and McFarland no 0.5‐1‐2‐3‐4). Among the tested isolates (including ATCC strain), 22 of them had DST results at increasing inoculum sizes that were 100% consistent with DST results in the reference inoculum and MGIT‐960. For alone streptomycin (STR), in seven isolates tested, DST results at increasing inoculum sizes were 100% consistent with MGIT‐960 results but were inconsistent with results determined with the reference inoculum. These isolates were found to be susceptible to STR with reference inoculum, but resistant to STR in MGIT‐960 and increasing inoculum sizes. Our study reveals that inoculum size does not affect DST results of *M. tuberculosis*; on the contrary, increasing the inoculum may have positive results for some antibiotics, such as STR.

## Graphical abstract

Impact of increased inoculum size on drug susceptibility testing of Mycobacterium tuberculosis and its role in the detection of drug resistance

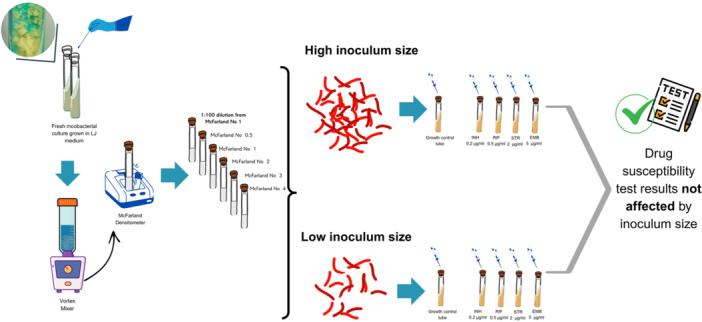

## 1 Introduction

The important part for drug susceptibility test (DST) of *Mycobacterium tuberculosis* isolates is the preparation stage of the bacterial inoculum. Tuberculosis (TB) bacilli have a rough (R‐type) colony morphotype known for increased cellular aggregation. TB bacilli in the R morphotype form compact colonies with cord‐like structures (Howard et al. 2006). In most TB studies, homogeneous mycobacterial suspensions containing isolated bacilli are used. For this, bacilli are either cultured in media containing Tween or subjected to physical disruption procedures (Brambilla et al. 2016). In order to obtain a homogeneous TB bacillus suspension, a physical disintegration procedure using sterile glass beads is applied in phenotypic DSTs. Following the homogenization stage, serial dilutions of the bacterial suspension are made to adjust the inoculum recommended for the test. This situation is quite worrying in terms of biosafety because aerosols containing bacilli are dispersed into the air and carry the risk of infection (World Health Organization 2018). The homogenization stage may be essential for the reproducibility of DSTs and the prevention of erroneous results, but it is unclear which aspect of the inoculum adjustment stage is critical. The “inoculum effect,” which can affect DST results, is widely mentioned in the literature for bacteria other than *M. tuberculosis*. It is estimated that this phenomenon is the reason behind the recommendation of a standard inoculum size for *M. tuberculosis* isolates (Jung et al. 2018). However, except for our study conducted in 2023 focusing on the relationship between DST results and inoculum size (Yildirim et al. 2023), no other recent study has been found. Although DST results and inoculum size attracted the attention of researchers in the years when only one or two anti‐TB agents were newly discovered, the findings obtained need to be supported and clarified (Youmans 1945; Williston and Youmans 1949; Robinson et al. 1950; Youmans et al. 1947). In this study, we investigated the effect of inoculum size of *M. tuberculosis* isolates with different primary drug susceptibility profiles on DSTs for primary drugs determined by the proportion method on Middlebrook 7H10 agar.

## 2 Methods

### 2.1 *M. tuberculosis* Strains

All strains tested in the study were obtained from the culture collection of Akdeniz University, Faculty of Health Sciences, Research Laboratory‐Tuberculosis Research Unit (Antalya, Turkey). Five reference ATCC strains and 24 *M. tuberculosis* isolates were tested. The resistance profiles of the tested isolates are shown in Table 1.

**Table 1:** Drug resistance profiles of *M. tuberculosis* isolates tested in this study (based on BACTEC MGIT 960 results).

Resistance profile	Number of isolates
Susceptible to all primary drugs	9
Resistant to INH	1
Resistant to RIF	1
Resistant to STR	3
Resistant to STR + INH	4
Resistant to EMB + INH	1
Resistant to INH + RIF	2
Resistant to INH + RIF + STR	2
Resistant to INH + RIF + EMB	1
*Total number*	**24**

Abbreviations: EMB, ethambutol; INH, isoniazid; RIF, rifampicin; STR, streptomycin.

### 2.2 Preparation of Mycobacterial Inoculum

Bacterial inoculums were prepared using fresh bacterial cultures grown in Lowenstein‐Jensen (LJ) medium, as previously recommended by Yildirim et al (Yildirim et al. 2023). All isolates were prepared according to McFarland no 0.5, 1, 2, 3 and 4 turbidity using a McFarland densitometer (BIOSAN Riga, Latvia). A 10‐2 dilution of McFarland no 1 turbidity was performed to be used as the reference inoculum (World Health Organization 2018).

### 2.3 Preparation of Antibiotic Solutions

In this study, isoniazid (INH), rifampicin (RIF), streptomycin (STR) and ethambutol (EMB) were tested as primary anti‐TB drugs. All antibiotics were used in powder form provided by the manufacturer (Sigma‐Aldrich, USA). INH, STR, and EMB were dissolved in sterile distilled water, and RIF was dissolved in methanol to prepare stock antibiotic solutions at a concentration of 8192 μg/mL.

### 2.4 Preparation of Middlebrook 7H10 Agar for the Proportion Method

Middlebrook 7H10 agar (BD Difco, USA) was prepared according to the manufacturer's recommendations. Antibiotic solutions were added to a final concentration of 0.2 μg/mL for INH, 0.5 μg/mL for RIF, 5 μg/mL for EMB and 2 μg/mL for STR and the media were distributed to 5 mL in screw‐capped sterile tubes. All tubes were left to solidify at room temperature in a slanted position, away from light. The prepared tubes were stored at + 4°C until the day of the study, and the storage period did not exceed 4 weeks (World Health Organization 2018).

### 2.5 Proportion Method on Middlebrook 7H10 Agar With Different Inoculums

**Figure 1:**
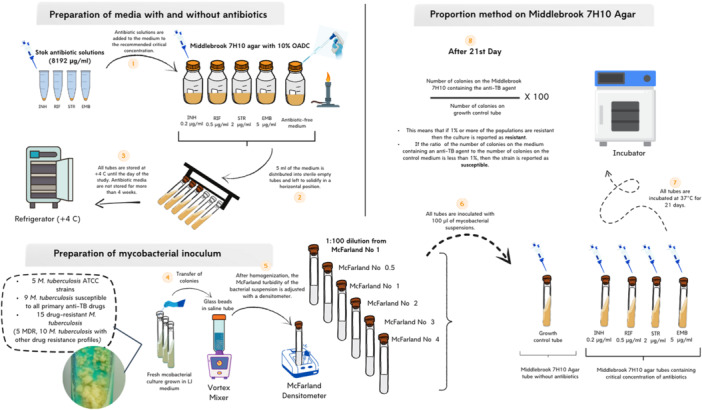
Implementation of the proportion method on Middlebrook 7H10 agar using different inoculum sizes.

The proportion method on 7H10 agar using different inocula is illustrated in Figure 1. One growth control tube without antibiotics and four antibiotic tubes containing critical concentrations of INH, RIF, STR, and EMB were prepared for all test isolates. These five‐tube experimental sets were prepared separately for six different inocula of each isolate in total (McFarland no 0.5, 1, 2, 3, 4 and 10^−2^ dilution of McFarland no 1). Bacterial inocula prepared as described in Section 2.2 were inoculated into the growth control tube and test tubes in 100 μL quantities. After inoculation, all tubes were incubated at 37°C for 21 days. At the end of the 21‐day incubation, if sufficient growth was observed in the growth control tube, the test was terminated. Drug susceptibility results were evaluated with a 1% proportion approach.

## 3 Results

In the study, the test was terminated on the 21st day of incubation for all inoculum sizes of all test isolates, and the results were evaluated using the 1% ratio approach. Among the tested inoculums, the 10^−2^ dilution of McFarland no 1 was accepted as the recommended reference inoculum and was used to compare the sensitivity results determined with other inoculums and BACTEC MGIT 960. DST results at all inoculum sizes of reference ATCC strains were 100% compatible with the reference inoculum size and BACTEC MGIT‐960 for all primary drugs (Table 2). The DST results of 9 *M. tuberculosis* isolates known to be susceptible to all primary drugs in increasing inoculum sizes were 100% compatible with the reference inoculum size and with BACTEC MGIT‐960 for all primary drugs (Table 3). Among these susceptible isolates, microcolonies were found in the tubes containing EMB of 3, and 4 McFarland inoculums of IST‐21 isolate, and in the tubes containing STR and EMB of 2 and 4 McFarland inoculums of IST‐5 isolate. However, the number of microcolonies was less than 1% of the number of colonies in the growth control tubes. Therefore, the categorical agreement of the isolates remained the same. The formation of microcolonies in the presence of EMB has been reported previously (Madison et al. 2002; Clinical and Laboratory Standards Instıtute. 2018). Among the 5 MDR‐TB isolates, the DST results of 3 isolates at increasing inoculum sizes were 100% compatible with the reference inoculum size and the BACTEC MGIT‐960 result. The other 2 isolates, C‐8 and MDR‐2, were resistant to INH‐RIF‐STR at all increasing inoculum sizes (compatible with BACTEC MGIT 960 results) and were observed to be resistant only to INH‐RIF in the reference inocula. With the decrease in inoculum size, STR resistance could not be detected in these 2 isolates, and a “false sensitive” result was obtained. It was observed that there was a discrepancy between the DST determined with the reference inoculum size recommended for the 7H10 agar proportion method and the BACTEC MGIT‐960 susceptibility results. In one of the isolates whose DST results were found to be compatible with the reference inoculum (2013‐3), microcolonies were found in the EMB tubes of 3 and 4 McFarland inoculums (Table 2). In 5 of the 10 isolates with different resistance profiles (C‐1, C‐3, C‐4, C‐6, A‐5), DST results at all increasing inoculum sizes were 100% compatible with the reference inoculum size and BACTEC MGIT‐960 results. In addition to INH resistance, 2 of these 5 isolates (C‐1 and C‐6) also had STR resistance, and as expected, colonies were observed in the STR tubes in the reference inoculum. In contrast, in all of the remaining 5 isolates with different resistance profiles (C‐2, C‐5, C‐10, C‐13, and C‐14), STR resistance was known to be present (compatible with BACTEC MGIT 960 results) and was observed in all increasing inoculum sizes, but STR resistance was not observed in the reference inoculum, resulting in inconsistency. In the primary drugs of these isolates, except STR, all inocula were 100% compatible with the reference inoculum. In 3 of these 10 isolates with different resistance profiles (C‐3, C‐4, C‐10), random, sparse colonies were found in RIF tubes regardless of inoculum size. This situation did not affect the categorical compatibility of the isolates. This situation was not observed in other RIF susceptible isolates (Table 4).

**Table 2:** Effect of inoculum size on primary drug susceptibilities of *M. tuberculosis* ATCC and MDR strains.

*M. tuberculosis*	DST results of different inoculums (INH*, RIF*, STR*, EMB*)					
	Reference inoculum	0.5 McF	1 McF	2 McF	3 McF	4 McF
ATCC 27294	SSSS	SSSS	SSSS	SSSS	SSSS	SSSS
ATCC 35822	RSSS	RSSS	RSSS	RSSS	RSSS	RSSS
ATCC 35838	SRSS	SRSS	SRSS	SRSS	SRSS	SRSS
ATCC 35820	SSRS	SSRS	SSRS	SSRS	SSRS	SSRS
ATCC 35837	SSSR	SSSR	SSSR	SSSR	SSSR	SSSR
C‐8	RRSS	RRRS	RRRS	RRRS	RRRS	RRRS
2013‐3	RRSS	RRSS	RRSS	RRSS	RRSS	RRSS
MDR‐1	RRSR	RRSR	RRSR	RRSR	RRSR	RRSR
MDR‐2	RRSS	RRRS	RRRS	RRRS	RRRS	RRRS
MDR‐4	RRSS	RRSS	RRSS	RRSS	RRSS	RRSS

*Note:* Reference inoculum; 10^−2^ dilution of McFarland no 1.

Abbreviations: EMB, ethambutol; INH, isoniazid; McF, McFarland standard; R, resistance; RIF, rifampicin; S, sensitive; STR, streptomycin.

**Table 3:** Effect of inoculum size on drug susceptibilities of primary drug‐susceptible *M. tuberculosis* isolates.

*M. tuberculosis*	DST Results of Different inoculums (INH*, RIF*, STR*, EMB*)					
	Reference inoculum	0.5 McF	1 McF	2 McF	3 McF	4 McF
IST‐11	SSSS	SSSS	SSSS	SSSS	SSSS	SSSS
IST‐18	SSSS	SSSS	SSSS	SSSS	SSSS	SSSS
IST‐9	SSSS	SSSS	SSSS	SSSS	SSSS	SSSS
IST‐21	SSSS	SSSS	SSSS	SSSS	SSSS	SSSS
IST‐6	SSSS	SSSS	SSSS	SSSS	SSSS	SSSS
IST‐5	SSSS	SSSS	SSSS	SSSS	SSSS	SSSS
AHS‐24	SSSS	SSSS	SSSS	SSSS	SSSS	SSSS
IST‐7	SSSS	SSSS	SSSS	SSSS	SSSS	SSSS
IST‐10	SSSS	SSSS	SSSS	SSSS	SSSS	SSSS

*Note:* Reference inoculum; 10^−2^ dilution of McFarland no 1.

Abbreviations: EMB, ethambutol; INH, isoniazid; McF, McFarland standard; R, resistance; RIF, rifampicin; S, sensitive; STR, streptomycin.

**Table 4:** Effect of inoculum size on drug susceptibilities of *M. tuberculosis* isolates with different resistance profiles.

*M. tuberculosis*	DST results of different inoculums (INH*, RIF*, STR*, EMB*)					
	Reference inoculum	0.5 McF	1 McF	2 McF	3 McF	4 McF
C‐1	RSRS	RSRS	RSRS	RSRS	RSRS	RSRS
C‐2	RSSS	RSRS	RSRS	RSRS	RSRS	RSRS
C‐3	RSSS	RSSS	RSSS	RSSS	RSSS	RSSS
C‐4	RSSR	RSSR	RSSR	RSSR	RSSR	RSSR
C‐5	SSSS	SSRS	SSRS	SSRS	SSRS	SSRS
C‐6	RSRS	RSRS	RSRS	RSRS	RSRS	RSRS
C‐10	RSSS	RSRS	RSRS	RSRS	RSRS	RSRS
C‐13	SSSS	SSRS	SSRS	SSRS	SSRS	SSRS
C‐14	SSSS	SSRS	SSRS	SSRS	SSRS	SSRS
A‐5	SRSS	SRSS	SRSS	SRSS	SRSS	SRSS

*Note:* Reference inoculum; 10^−2^ dilution of McFarland no 1.

Abbreviations: EMB, ethambutol; INH, isoniazid; McF, McFarland standard; R, resistance; RIF, rifampicin; S, sensitive; STR, streptomycin.

## 4 Discussion

The three main components in the application of DST are the medium, antimicrobial agent and test microorganism. All three of these can affect the DST results individually. This study focused on the effects of the amount of the test microorganism on DST. It is recommended that DSTs be performed using inocula containing a homogeneous suspension of the test microorganism. The size of the inoculum varies depending on the method to be applied and the type of bacteria and is an element that needs to be standardized. It is mandatory to use a standard inoculum for error‐free and repeatable DST results (Kim 2005). In particular, the inoculum preparation stage should be carried out carefully in species that are prone to clustering, such as *M. tuberculosis* isolates with the R morphology. Because insufficiently homogenized suspensions may produce indeterminate colony numbers per ml, which may lead to erroneous DSTs (Howard et al. 2006). It is noteworthy that the inoculum size varies according to the type of DST applied for *M. tuberculosis*. In DSTs in solid media (mostly proportion method), 10^−2^ or 10^−4^ dilutions of McFarland no 1, in the E‐Test method, which is not very frequently preferred, direct ≥ 3 McFarland turbidity, and in phenotypic colorimetric tests, 1:20, 1:10, 1:5 dilutions of McFarland no 1 are used as inoculum sources, again varying depending on the type of test (Clinical and Laboratory Standards Institute 2018; Biodisk 2005; Clinical and Laboratory Standards Institute 1995; Coban et al. 2015). Although such a variety of inoculums has brought to mind the possibility that inoculum size may affect DST results, studies in the literature do not prove this. Although studies explaining the relationship between DST and inoculum size for *M. tuberculosis* attracted the attention of researchers between 1945 and 1955, when primary anti‐TB drugs were newly discovered, these studies were not continued. Although a recent study by our team attempted to shed light on this issue, it was stated that more studies were needed (Yildirim et al. 2023). In the first studies on the subject, researchers reported that inocula of 0.1–1.0 mg, 0.01–0.25 mg, and 0.1–3.0 mg sizes did not affect the STR sensitivity of *M. tuberculosis*, respectively (Youmans 1945; Williston and Youmans 1949; Robinson et al. 1950). Unlike STR, it has been reported that the DST of paraamino salicylic (PAS) is affected by the number of bacteria in the inoculum (Youmans et al. 1947). In another study in 1955, it was reported that the inoculum size, which included 2.5–3.7 mg of fresh TB bacilli and its dilutions from 10^−1^ to 10^−7^, affected the apparent sensitivities of STR, INH and PAS (Kenney et al. 1955). In those years, other anti‐TB drugs had not yet been discovered or their activities on TB had not been determined; therefore, the available studies on the subject were limited to these. It is known today that only the homogenization stage of inoculum preparation can have an effect on DST results in *M. tuberculosis (*Mitchison 2005 *)*. Apart from this, the effect of the inoculum adjustment stage, where the inoculum is diluted, on DST results is still unclear. Although molecular techniques are increasingly used for resistance detection, conventional phenotypic DST methods remain the most accessible and routinely applicable approaches in many tuberculosis‐endemic and resource‐limited settings. Therefore, optimization and standardization of methodological variables such as inoculum preparation and homogenization are still critically important for obtaining reliable susceptibility results.

In our study, we showed the effect of inoculum size on drug susceptibility results of *M. tuberculosis* isolates with different susceptibilities using the proportion method, which is a generally accepted method in terms of standardization among culture‐based methods. In our study, which we carried out using Middlebrook 7H10 agar, we tested five reference ATCC strains, nine susceptible to all primary drugs, five MDR‐TB and 10 *M. tuberculosis* isolates with different resistance profiles. The recommended reference inoculum size is 10^−2^ dilution of McFarland no 1, and tests were performed for each isolate in five different inocula (McFarland no 0.5‐1‐2‐3‐4). DST results in all increasing inocula of five ATCC strains and 17 isolates of *M. tuberculosis* were found to be 100% compatible with the reference inoculum and BACTEC MGIT‐960 results. Two MDR isolates and five isolates with different resistance profiles were found to be STR resistant in all increasing inocula, in line with the BACTEC MGIT‐960 result, while STR was recorded as sensitive in the reference inoculum. The agreement with the reference inoculum was found to be 100% for all drugs except STR. The recommended reference inoculum size was not sufficient to detect STR resistance in the proportion method, and a higher inoculum had to be used. STR resistance is dependent on the presence of mutants seen in approximately 1 in 2.95 × 10^−8^ bacteria (Palomino et al. 2007). Therefore, at low inoculum density, some resistant mutants in the population may not have been selected, and this may show “falsely sensitive” results. It is also known that there is lower agreement between the proportion method and BACTEC MGIT‐960 for STR and EMB compared to INH and RIF. On the other hand, it is known that it is difficult to measure STR and EMB resistance consistently and reliably due to the technical limitations of the methods (Said et al. 2012; Giampaglia et al. 2007). Mycobacterial populations are quite heterogeneous and consist of subpopulations with different phenotypic characteristics. Heterogeneity is a survival strategy against dynamic and variable stress factors during infection. Different subpopulations also show diversity in drug responses (Chung et al. 2022). It has recently been shown that even within the same cluster, bacilli may show different levels of resistance to STR (Rocha et al. 2021). For these reasons, it may be useful to use a high inoculum size to reveal resistant mutants in the population or the critical concentration of the drug may need to be re‐standardized. Our results suggest that revisiting traditional methodological parameters may improve the accuracy of phenotypic DST results in routine microbiology laboratories, potentially contributing to more effective patient management and reduction of multidrug‐resistant tuberculosis.

In our study, while investigating the effect of increasing the inoculum size used in the proportion method on DST results, we obtained data showing the incompatibility between the proportion method and BACTEC MGIT‐960. The proportion method failed to reveal resistant subpopulations for STR and to detect resistance according to the presence of visible colonies compared to BACTEC MGIT‐960. The success of BACTEC MGIT‐960 in revealing low‐level STR resistance stems from the technical advantage of the method. In the method, as a principle, fluorometric evaluation is made as a result of the number of bacilli showing a logarithmic increase over time in liquid culture exceeding a certain threshold value. In this case, even the few resistant bacilli will give positive results above a certain value with logarithmic increase. Therefore, liquid cultures are considered to be more sensitive compared to solid cultures. However, in the proportion method, the number of bacilli inoculated into the solid medium will be equal to the number of colonies formed. The chance of detecting the few resistant bacilli that cause low‐level resistance in the population increases only with increasing the inoculum size. As in our DST results, the existence of isolates that are “STR‐sensitive” in the proportion method but “STR‐resistant” in BACTEC MGIT‐960 has been reported in other studies (Said et al. 2012; Giampaglia et al. 2007). In order to achieve consistency between DSTs, both the cc of the drugs and the recommended inoculum concentrations need to be reviewed.

In our study, in two test isolates (IST‐21 and 2013‐3), microcolonies were found only in the medium containing EMB in McFarland no 3 and 4 turbidities with increasing inoculum size. The presence of microcolonies has been reported both in our previous study (Yildirim et al. 2023) and in other studies (Clinical and Laboratory Standards Institute 2018). In one of the test isolates, low levels of microcolonies were found in both STR and EMB‐containing media in McFarland no 2 and no 4 turbidities. The presence of microcolonies was below 1% for all 3 isolates and did not change the categorical agreement of the isolates. As the inoculum size increases, the probability of encountering microcolonies also increases. However, thanks to the high inoculum, both low‐level drug resistance can be shown and colonies can be seen with the eye in a shorter time with the increase in bacillus density.

In our study, the other antibiotic that we thought was affected by the heterogeneity of the bacillus population was RIF. Although it did not change the categorical agreement of the isolates, visible colonies were detected in 3 of the RIF‐susceptible isolates.

These growths were not directly proportional to the increase in inoculum; on the contrary, they were coincidental. This situation may be due to the heterogeneity of the population. Among all the test isolates, including ATCC strains, it is known that all 22 isolates are susceptible to RIF, but these coincidental colonies were observed in only 3 of them, and in all the rest, the RIF antibiotic showed its sterilizing effect. One of the factors affecting this situation may be the revised critical concentration (cc) of the RIF antibiotic. In 2021, the cc of RIF antibiotic in 7H10 and MGIT 960, which was 1 μg/ml, was reduced to 0.5 μg/mL in order to detect borderline resistant isolates and prevent false RIF sensitivity (World Health Organization 2021). However, if there was a resistance that emerged with the decrease in concentration, it could be expected that a similar situation would be seen in other RIF susceptible isolates or that the growth in the medium containing RIF would increase proportionally with the increase in inoculum. The most reasonable justification seems to be the complex heterogeneity of the population. Genetic mutations may not always be effective due to heterogeneity in the population. As in phenotypic heterogeneity, a significant phenotypic resistance to RIF can be observed as a result of faulty translation (Dhar et al. 2016; Javid et al. 2014). More important than the effect of inoculum size on DST is the ratio of the susceptible or resistant subpopulation in the bacillus population. For this, analyses are performed at the single‐cell level, but this is not possible for routine DSTs (Chung et al. 2022; Pisu et al. 2021). In addition to technical factors, strain‐specific genetic differences and molecular resistance mechanisms may contribute to variability in testing susceptibility, highlighting the need for complementary molecular investigations.

Drug resistance in *M. tuberculosis* is not a homogeneous response mechanism by any means, but rather is quite heterogeneous. Moreover, none of the methods developed to detect resistance is perfect; on the contrary, they have difficulty in finding harmony with each other (Böttger 2011). Our study explained the effects of inoculum size on DST, while also showing the advantages of using high inoculum density and the limitations of using low inoculum density. The homogenization stage of inoculum preparation is important only in terms of DST results and reproducibility of tests, not because of the inoculum effect. Therefore, it is a stage that should not be skipped. However, if the inoculum adjustment and dilution stages are eliminated at this stage, which is quite worrying in terms of biosafety, the risk of infection will be reduced. Future studies involving larger numbers of clinical isolates and different *M. tuberculosis* strain types are needed to establish optimized homogenization protocols and evaluate their impact on DST reproducibility. Although no contamination events were observed in the present study, larger‐scale studies including more patient‐derived isolates are necessary to better evaluate contamination risk and biosafety considerations associated with increased inoculum densities.

## 5 Conclusions

Increasing inoculum size did not adversely affect DST results and often aligned with BACTEC MGIT‐960. Minor discrepancies highlight the need for standardization and awareness of methodological differences, especially in low‐level resistant *M. tuberculosis* isolates.

## Author Contributions

**Kubra Yildirim:** conceptualization, investigation, methodology, validation, writing – review and editing. **Cemilenur Atas:** investigation, writing – review and editing, visualization, data curation. **Ece Simsek:** investigation, methodology, conceptualization, writing – review and editing, supervision, validation. **Mir Pooya Salehi Moharer:** writing – review and editing, data curation, investigation. **Meltem Uzun:** conceptualization, investigation, methodology, validation, writing – original draft, supervision, project administration. **Ahmet Yilmaz Coban:** conceptualization, investigation, methodology, validation, writing – original draft, project administration, supervision.

## Ethics Statement

The authors have nothing to report.

## Consent

The authors have nothing to report.

## Conflicts of Interest

The authors declare no conflicts of interest.

## Data Availability

Data sharing not applicable to this article as no datasets were generated or analyzed during the current study.
